# 3D Monte Carlo bone marrow dosimetry for Lu-177-PSMA therapy with guidance of non-invasive 3D localization of active bone marrow via Tc-99m-anti-granulocyte antibody SPECT/CT

**DOI:** 10.1186/s13550-019-0548-z

**Published:** 2019-08-14

**Authors:** Astrid Gosewisch, Harun Ilhan, Sebastian Tattenberg, Andrea Mairani, Katia Parodi, Julia Brosch, Lena Kaiser, Franz Josef Gildehaus, Andrei Todica, Sibylle Ziegler, Peter Bartenstein, Guido Böning

**Affiliations:** 1 Department of Nuclear Medicine, University Hospital, LMU Munich, Marchioninistrasse 15, 81377 Munich, Germany; 2 Heidelberg Ion Beam Therapy Center, University Hospital Heidelberg, Heidelberg, Germany; 3 Department of Medical Physics, Ludwig-Maximilians-Universität München, Garching b. München, Germany

**Keywords:** Radioligand therapy, Dosimetry, Monte Carlo, Bone marrow, mCRPC, Lutetium, PSMA, Tc-99m-anti-granulocyte antibody scintigraphy, Bone marrow localization

## Abstract

**Background:**

The bone marrow (BM) is a main risk organ during Lu-177-PSMA ligand therapy of metastasized castration-resistant prostate cancer (mCRPC) patients. So far, BM dosimetry relies on *S* values, which are pre-computed for reference anatomies, simplified activity distributions, and a physiological BM distribution. However, mCRPC patients may show a considerable bone lesion load, which leads to a heterogeneous and patient-specific activity accumulation close to BM-bearing sites. Furthermore, the patient-specific BM distribution might be significantly altered in the presence of bone lesions. The aim was to perform BM absorbed dose calculations through Monte Carlo (MC) simulations and to investigate the potential value of image-based BM localization.

This study is based on 11 Lu-177-PSMA-617 therapy cycles of 10 patients (10 first cycles), who obtained a pre-therapeutic Ga-68-PSMA-11 PET/CT; quantitative Lu-177 SPECT acquisitions of the abdomen 24 (+CT), 48, and 72 h p.i.; and a Lu-177 whole-body planar acquisition at 24 h post-therapy. Patient-specific 3D volumes of interest were segmented from the Ga-68-PSMA-11 PET/CT, filled with activity information from the Lu-177 data, and imported into the FLUKA MC code together with the patient CT. MC simulations of the BM absorbed dose were performed assuming a physiological BM distribution according to the ICRP 110 reference male (MC1) or a displacement of active BM from the direct location of bone lesions (MC2). Results were compared with those from *S* values (SMIRD). BM absorbed doses were correlated with the decrease of lymphocytes, total white blood cells, hemoglobin level, and platelets. For two patients, an additional pre-therapeutic Tc-99m-anti-granulocyte antibody SPECT/CT was performed for BM localization.

**Results:**

Median BM absorbed doses were 130, 37, and 11 mGy/GBq for MC1, MC2, and SMIRD, respectively. Significant strong correlation with the decrease of platelet counts was found, with highest correlation for MC2 (MC1: *r* = − 0.63, *p* = 0.04; MC2: r = − 0.71, *p* = 0.01; SMIRD: *r* = − 0.62, *p* = 0.04). For both investigated patients, BM localization via Tc-99m-anti-granulocyte antibody SPECT/CT indicated a displacement of active BM from the direct location of lesions similar to model MC2 and led to a reduction in the BM absorbed dose of 40 and 41% compared to MC1.

**Conclusion:**

Higher BM absorbed doses were observed for MC-based models; however, for MC2, all absorbed doses were still below 2 Gy. MC1 resulted in critical values for some patients, but is suspected to yield strongly exaggerated absorbed doses by neglecting bone marrow displacement. Image-based BM localization might be beneficial, and future studies are recommended to support an improvement for the prediction of hematoxicities.

## Introduction

In radioligand therapy, dosimetry is recommended for appropriate treatment planning and aims for maximizing the absorbed dose to malignant structures, while minimizing the absorbed dose to risk organs. A variety of strategies for the assessment of tissue absorbed doses exist, which can be distinguished depending on how detailed the patient-specific information is considered or depending on whether a 3D-absorbed dose model is used or absorbed dose factors (*S* values) are applied [1–5]. Monte Carlo techniques can be used to fully simulate all interactions of radioactive decay particles within the surrounding material in a step-by-step manner. Various Monte Carlo codes such as FLUKA or GEANT4 were extended to applications in nuclear medicine and are capable to consider the patient-specific 3D activity and anatomical characteristics via inclusion of the SPECT, PET, and CT data into the simulation [6–12]. Thus, 3D-absorbed dose distributions with resolution and accuracy depending on the input image data can be provided. However, the absorbed dose to risk organs or tumors during radioligand therapy is usually calculated via organ-level *S* values, which were pre-calculated based on standardized anthropomorphic phantoms and which estimate the mean absorbed dose to the whole target region based on the mean time-integrated activity in a specified source region.

The active bone marrow (BM) is a main organ at risk during Lu-177-PSMA ligand therapy, especially as patients with advanced mCRPC often present with a high bone tumor burden and a potentially reduced hematological function [13–15]. Typically, bone marrow dosimetry is performed by applying the aforementioned *S* values and accounts for the self-absorbed dose to the bone marrow from the blood, the cross-absorbed dose from the remainder of the body (ROB), and the cross-absorbed dose from major organs and tumors as specific source regions [15–19]. However, during bone marrow dosimetry using organ-level *S* values, simplifying assumptions have to be made to estimate the absorbed dose from the overall tumor distribution, as no pre-calculated *S* values exist that consider all lesions in their size, shape, number, and location. Thus, Monte Carlo simulations may lead to improved bone marrow absorbed dose estimates, as they have the potential to fully account for the patient-specific 3D disease characteristics.

Another limitation of classical bone marrow dosimetry is that the actual localization of the active bone marrow is a priori unknown. Bone lesions might lead to a displacement of active bone marrow from the direct site of metastases and thus activity accumulation, which would drastically reduce the absorbed dose to the active bone marrow [20, 21]. However, bone marrow dosimetry using pre-calculated organ-level *S* values assumes a physiological bone marrow distribution [16]. Clinical imaging methods, such as magnetic resonance imaging or Tc-99m-anti-granulocyte antibody scintigraphy, can be used for non-invasive active bone marrow localization, within the spatial resolution of the corresponding imaging modality [22–25]. Thus, such techniques might overcome the limitation of an a priori unknown target region for bone marrow dosimetry.

In this study, we performed 3D simulations of the bone marrow absorbed dose for mCRPC patients, assuming either an active bone marrow distribution, which is not altered by the bone tumor load, or a displacement of active bone marrow from the location of bone metastasis. These results were compared to the respective bone marrow absorbed dose estimates derived via organ-level *S* values. Subsequently, all absorbed dose estimates were further correlated with the patient-specific changes in hematological parameters. For a subgroup of investigated patients, a Tc-99m-anti-granulocycte antibody SPECT/CT was acquired prior to therapy, which was further analyzed to investigate Monte-Carlo-based bone marrow dosimetry with and without knowledge of the patient-specific active bone marrow distribution.

If not indicated otherwise, the term bone marrow always refers to the active bone marrow, which represents the radio-sensitive part of the overall bone marrow mixture.

## Material and methods

### Patients and data acquisition

This study is based on the first cycle of 10 patients, who showed PSMA avid soft tissue and bone lesions on the pre-therapeutic whole-body Ga-68-PSMA-11 PET/CT. Patients P1–P4 were treated with on average 3.7 GBq Lu-177-PSMA-617, while for all other patients, the dosing scheme was increased to 6 GBq, according to initial dosimetry results at our institution [15, 26] (Table 1). Patient P7 received a fifth therapy cycle at our institution with prior image-based active bone marrow localization and was included as patient P8 (Table 1), as this rare data allows for an exemplarily comparison of patient-specific bone marrow dosimetry without and with knowledge of the patient-specific bone marrow distribution. All patients received a 15-min abdominal Lu-177 SPECT/CT scan and a 20-min whole-body planar scintigraphy at 24 h p.i., as well as a 15-min abdominal SPECT at 48 h and 72 h p.i. on a dual-headed Symbia T2 SPECT/CT (Siemens Medical Solutions, Erlangen, Germany). The SPECT and planar whole-body acquisitions were based on a standard Lu-177 imaging protocol, using a medium-energy low-penetration collimator, the photopeak at 208 keV (width 15%), and two additional scatter windows at 170 keV (width 15%) and 240 keV (width 10%) [15, 26, 27]. For dosimetry purposes, five venous blood samples were drawn from each patient 30 and at maximum 80 min post-start of infusion, and before each image acquisition. Blood parameters were further monitored as a part of clinical routine until the next therapy cycle [28]. Monitoring of blood element counts was performed at the morning prior to therapy (baseline), each morning during the following 3 days until discharge, and at 4 and 8 weeks until the next therapy cycle.

**Table 1:** Characteristics of mCRPC patients included in this study

	P1	P2	P3	P4	P5	P6	P7	P8	P9	P10	P11
Age	66	68	47	61	73	63	82	83	79	67	88
Activity (GBq)	3.7	3.7	3.7	3.7	6.0	6.0	6.0	6.0	6.0	6.1	6.1
Cycle	1	1	1	1	1	1	1	5	1	1	1
Metastases (VIS = visceral, LYM = lymph, OSS = osseous)	OSS	Mainly VIS (liver), OSS, LYM	Mainly OSS, LYM	Mainly OSS, LYM	Mainly OSS, LYM	OSS, LYM	Mainly OSS, LYM, VIS	Mainly OSS, LYM, VIS	Mainly OSS, LYM	Mainly OSS, LYM	OSS
Initial TNM classification and Gleason score	pT4, N1, R1, G3, Gleason 8	pT3b, pN1, R0, G3, Gleason 9	pT3a, pN1, pR1, Gleason 9	pT3b, pN1, R1, Gleason 9	pT3b, pN1	T3a, N1	pT4, pN1, R1, G3, Gleason 9	pT4, pN1, R1, G3, Gleason 9	n.a.	pT3, pN0, R1, Gleason 7	n.a.
PSA (ng/ml) prior to RLT	1201	368	408	5436	2311	0.86	65.7	65.6	418	20.6	52.1
Pre-therapies (1, yes/0, no)											
▪ Surgery	1	0	1	1	1	1	1	1	1	1	0
▪ Radiotherapy	1	1	0	1	1	0	1	1	0	1	0
▪ Anti-hormonal therapy (including 2nd line anti-hormonal therapy with bicalutamide, enzalutamide, abiraterone acetate)	1	1	1	1	1	1	1	1	1	1	1
▪ Ra-223	1	0	1	0	0	0	0	0	0	1	0
▪ Chemotherapy (docetaxel, cabazitaxel)	1	1	1	1	1	1	0	0	1	1	0
Blood baseline											
▪ Leukocytes (G/l)	7.2	4.9	6	5.6	5.2	4.54	5.41	4.85	5.31	2.73	7.84
▪ Lymphocytes (G/l0	0.57	0.96	1.71	0.49	0.56	1.14	0.95	0.48	0.94	0.28	2.06
▪ Hemoglobin (g/dl)	14.1	12.3	12.8	9.6	10.9	13.3	10.5	8.4	10.5	8.3	12.1
▪ Thrombocytes (G/l)	195	307	323	291	235	227	450	305	223	89	281
▪ Erythrocytes (T/l)	4.79	4.31	4.36	4.08	3.86	4.11	3.96	3.25	3.55	3.2	3.64

For patients P8 and P9, an additional Tc-99m-anti-granulocyte antibody SPECT/CT (Scintimun, GLYCOTOPE Biotechnology GmbH, Heidelberg, Germany) was acquired prior to radioligand therapy for localization of the active bone marrow. A 25-min two-bed SPECT/CT scan of thorax and abdomen was performed at 3–4 h after the injection of approximately 400 MBq. Image acquisition followed a standard Tc-99m protocol using a low-energy high-resolution collimator, a photopeak window of 140 keV (width 15%), and an additional scatter window at 115 keV (width 20%).

All patients gave written consent to undergo radioligand therapy. The study protocol was approved by the local ethics committee of the Medical Faculty of the Ludwig-Maximilians-University Munich, which waived the necessity for written consent for study entry, as the study is based on retrospective and irreversibly anonymized patient data.

### Image reconstruction and quantification

Quantitative SPECT reconstruction was performed with an in-house maximum-a-posteriori reconstruction algorithm, which considers the correction for photon attenuation based on the co-registered low dose attenuation correction CT (AC-CT) (PMOD Version 3.609 rigid-body co-registration), correction for photon scattering based on the triple-energy-window (TEW) or dual-energy-window (DEW) method, and compensation of distance-dependent detector resolution using a Gaussian detector response model. Final quantification was realized by applying a system-specific calibration factor, which was determined from an identically imaged and reconstructed cylinder phantom of homogeneous and known activity concentration [15, 27, 29].

Quantitative reconstruction of Ga-68-PSMA-11 PET/CT scans was conducted as part of clinical routine using the TrueX algorithm with 3 iterations, 21 subsets, and a 3D post-reconstruction Gaussian filter with a full-width half maximum of 2 mm. The voxel volume in the PET data was 4.1 × 4.1 × 5.0 mm^3^.

All Lu-177 planar whole-body images were corrected for photon attenuation and scattering pixel by pixel via an in-house MATLAB routine. Correction of photon attenuation was achieved via a patient-specific *μ*-map, which was generated from the whole-body CT of the pre-therapeutic Ga-68-PSMA-11 PET/CT acquisition, while the correction of photon scattering employed the TEW method. A patient-specific calibration factor was determined utilizing a cross-calibration with the corresponding quantitative SPECT at 24 h post-injection [28].

### Reference bone marrow dosimetry using mass-scaled organ-level *S* values

Bone marrow dosimetry utilizing phantom-based organ-level *S* values was selected as reference method in this work. It considered the blood, both kidneys, and the remainder of body (ROB) as specific source regions [15–19, 28]. The respective *S* values of the RADAR standardized male anthropomorphic phantom were used [30]. All *S* values were scaled to the patient-specific anatomical conditions. The time-integrated activities for the blood and both kidneys were determined based on a bi-exponential and a mono-exponential fit to the available time-activity measurements, respectively. For determination of the blood-to-bone marrow absorbed dose, we employed a hematocrit-based red marrow-to-blood activity concentration ratio (RMBLR), as we assume no specific binding to bone marrow or blood cells [16, 28, 31]. This assumption results in a reduced RMBLR compared to a RMBLR of one, which is typically employed for Lu-177 PRRT.

The derivation of the time-integrated activity of the ROB from a single planar whole-body scan was achieved by applying a suitable hybrid SPECT-planar model, which has been investigated in a previous study [28]. Briefly, a mono-exponential curve was fitted to the total SPECT activity over time, and this curve was scaled with the whole-body activity at 24 h p.i. afterwards to estimate the patient-specific whole-body time-activity curve. After integration over time, kidney and blood time-integrated activities were subtracted to obtain the number of decays within the ROB compartment [16]. As the patient-specific lesion distribution was highly variable for the patients investigated in this study, no pre-calculated *S* values were available, which consider all lesions in their size, shape, number, and location. Thus, the consideration of the metastases and their non-negligible activity uptake within the *S* value method was achieved by including the total lesion activity within the ROB compartment.

In a previous study, the contribution from the ROB including the lesion activities was found to be the dominating component of the total bone marrow absorbed dose [28]. However, the patient-specific lesion distribution is assumed to be only inadequately considered via classical *S* value dosimetry. To investigate differences between Monte-Carlo-based and classical *S* value bone marrow dosimetry, all bone marrow absorbed doses were correlated to the total bone lesion load, the time-integrated tumor uptake, and the time-integrated ROB retention (MATLAB Pearson’s correlation). The total bone lesion load was obtained from the pre-therapeutic Ga-68-PSMA-11 PET via PMOD kmeans segmentation (PMOD Version 3.609), while time-integrated tumor uptake and ROB retention were derived by integrating both the mono-exponential ROB and lesion time-activity curves. To obtain a total-body lesion time-activity curve from the sequential abdominal SPECT, the assumption that the kinetics of all abdominal lesions is equivalent to that of all lesions throughout the patient body was made. The total-body lesion time-activity curve was then derived from all segmented tumors within the abdominal sequential SPECT (kmeans segmentation, PMOD Version 3.609), scaled with the ratio of total-to-abdominal lesion load.

### Monte Carlo simulation of bone marrow absorbed dose

For each patient, the absorbed dose to the bone marrow was simulated using the FLUKA MC code, which has been extended and validated for applications in nuclear medicine, and which is capable to include the patient-specific 3D anatomical and activity imaging data [6, 11, 12, 32].

#### Anatomical and activity simulation data

The pre-therapeutic diagnostic CT from the Ga-68-PSMA-11 PET/CT scan served as patient-specific whole-body anatomical map during the Monte Carlo simulations. Therefore, the CT was converted to a voxel-wise map of density and anatomical composition as described by Botta et al. and as required by the MC code [6]. The patient-specific and time-dependent 3D whole-body Lu-177 activity distribution was described by combining the information contained in the sequential Lu-177 SPECT, the Lu-177 whole-body planar scintigraphy, and the whole-body PET/CT data. First, a patient-specific 3D whole-body volume of interest (VOI) map was generated by segmenting the kidneys, the tumors, and the ROB in the PET/CT volume. Using the sequential Lu-177 SPECT and the single Lu-177 whole-body planar scintigraphy, the activity in these compartments was assessed for each time point 24, 48, and 72 h post-injection. Therefore, kidney VOIs and the overall tumor load were semi-automatically segmented (kmeans segmentation, PMOD Version 3.609) from both the quantitative SPECT scan 24 h p.i. and the Ga-68-PSMA-11 PET. The segmented kidney VOIs were confirmed by visual comparison with the AC-CT and the diagnostic CT, respectively. Both, the 24-h-based SPECT kidney and tumor VOIs were then manually registered to the following imaging days 48 h and 72 h post-administration. The time-integrated activity per voxel was determined for both compartments, to directly assign a total number of decays per voxel to each VOI of the patient-specific activity template. Thereby, the abdominal lesion time-integrated activity was multiplied with the ratio of total-to-abdominal lesion volume to estimate the total lesion time-integrated activity from the sequential abdominal SPECT, as already described. The ROB time-integrated activity was defined via the mentioned hybrid SPECT-planar model [28], while the ROB VOI itself was derived from a VOI outlining the whole CT volume from the PET/CT data.

#### Absorbed dose to the bone marrow

For most of the skeletal sites, the bone marrow shows a heterogeneous microstructure composed of small marrow cavities containing a composition of active bone marrow (BM) and inactive bone marrow (iaBM), with these cavities being separated by the spongiosa, i.e., small ridges of hard bone (HB). This highly heterogeneous microstructure is not visible on routine clinical imaging modalities. Thus, for simulation and estimation of the bone marrow absorbed dose, we implemented a weighting factor-based model into the FLUKA code, similar to the two-factor mass-energy absorption coefficient method described by Lee et al. [33]. It estimates the absorbed dose to the active bone marrow from the absorbed dose simulated within the total bone mixture, multiplied via a weighting factor *w*, which describes an effective interaction probability within the active bone marrow. This weighting factor can be calculated from the particle energy *E* of the dose-depositing photons or electrons; the fractions of active bone marrow, inactive bone marrow, and hard bone (*f*_BM_, *f*_iaBM_, and *f*_HB_, respectively) present in the skeletal region of interest; the photon mass attenuation coefficients ($\frac{\mu }{\rho }$) or the electron mass stopping powers ($\frac{S}{\rho }$) of the skeletal constituents:

1.1 $$ {D}_{\mathrm{BM}}={D}_{\mathrm{bone}}\cdotp w, $$

1.2 $$ w=\frac{\frac{\mu_{\mathrm{BM}}(E)}{\rho_{\mathrm{BM}}}}{\frac{\mu_{\mathrm{bone}}(E)}{\rho_{\mathrm{bone}}}}\ \mathrm{or}\ w=\frac{\frac{S_{\mathrm{BM}}(E)}{\rho_{\mathrm{BM}}}}{\frac{S_{\mathrm{bone}}(E)}{\rho_{\mathrm{bone}}}}, $$

1.3 $$ {\displaystyle \begin{array}{l}{\mu}_{bone}(E)={f}_{BM}\cdot \frac{\mu_{BM}}{\rho_{BM}}+{f}_{iaBM}\cdot \frac{\mu_{iaBM}(E)}{\rho_{iaBM}}+{f}_{\mathrm{HB}}\cdot \frac{\mu_{\mathrm{HB}}(E)}{\rho_{\mathrm{HB}}}\cdot \mathrm{or}\\ {}{S}_{\mathrm{bone}}(E)={f}_{\mathrm{BM}}\cdot \frac{S_{\mathrm{BM}}(E)}{\rho_{\mathrm{BM}}}+{f}_{\mathrm{iaBM}}\cdot \frac{S_{\mathrm{iaBM}}(E)}{\rho_{\mathrm{iaBM}}}+{f}_{\mathrm{HB}}\cdot \frac{S_{\mathrm{HB}}(E)}{\rho_{\mathrm{HB}}},\end{array}} $$

1.4 $$ {f}_{\mathrm{BM}}=\frac{m_{\mathrm{BM}}}{m_{\mathrm{bone}}}\ \mathrm{and}\ \mathrm{in}\ \mathrm{general}\ {f}_i=\frac{m_i}{m_{\mathrm{bone}}},i\in \left\{\mathrm{BM},\mathrm{iaBM},\mathrm{HB}\right\}, $$

1.5 $$ {f}_{\mathrm{BM}}+{f}_{\mathrm{iaBM}}+{f}_{\mathrm{HB}}=1. $$

**Fig. 1:**
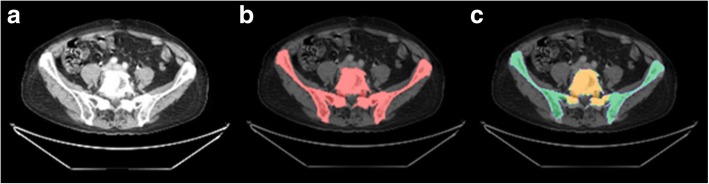
Exemplary workflow for definition of skeletal compositions. **a** Exemplary patient CT. **b** Semi-automatic segmentation of patient-specific bone VOI. **c** Automatic definition of bone regions from non-rigidly co-registered template

*f*_BM_, *f*_iaBM_, and *f*_HB_ reference values were available for 13 active-marrow-bearing bone regions according to the ICRP 110 reference male [34]. For each patient, these 13 bone regions were segmented onto the patient-specific diagnostic CT from the Ga-68-PSMA-11 PET/CT, and the aforementioned reference fractions *f*_BM_, *f*_iaBM_, and *f*_HB_ were assigned to each voxel according to its region affiliation. To facilitate segmentation of these 13 regions for each patient, a bone region template was employed. Therefore, the whole skeleton of 5 patients was segmented via a HU threshold of 200 on the diagnostic CT from the Ga-68-PSMA-11 PET/CT, and the remaining holes in these skeletal VOIs were manually filled afterwards (PMOD Version 3.609). For template generation, the segmented bone VOIs from all five patients were co-registered onto each other using PMOD non-rigid co-registration. Finally, all 13 regions were manually segmented on this template. For each patient, the whole skeleton was segmented in the same manner as for template generation, and the region-specific template was non-rigidly co-registered onto each patient-specific skeletal VOI, to automatically define both the patient-specific bone region classification and the related active bone marrow distribution containing reference values *f*_BM_ (Fig. 1).

This pre-defined active bone marrow distribution was used in two ways during Monte-Carlo-based bone marrow dosimetry. For model MC1, the active bone marrow distribution remained unchanged, assuming a co-localization of lesions and active bone marrow. For the second model MC2, all previously segmented lesion-containing voxels were removed from the active bone marrow distribution, to simulate the effect of a complete bone marrow displacement from the metastatic sites onto the absorbed dose estimates.

As for SMIRD, absorbed dose estimates were correlated to the bone lesion load, the time-integrated tumor uptake, and the time-integrated ROB retention (MATLAB Pearson’s correlation).

#### Simulation

During simulation, photon production, and transport thresholds were set to 1 keV (mean range < 1 mm for all tissues [35]). The corresponding electron thresholds were chosen as 10 keV (mean range < 0.01 mm for all tissues). 10^9^ decays were simulated for each simulation study.

### Correlation with blood parameters

For the patients included in this study, all bone marrow absorbed doses were correlated (MATLAB Pearson’s correlation) with the change of hematological parameters after the investigated therapy cycle (Table 1), i.e., the ratio of nadir-to-baseline values of platelet, lymphocyte, and leukocyte counts as well as of hemoglobin level. Particularly, the goal was to assess for the overall patient cohort whether patient-specific Monte-Carlo-based bone marrow dosimetry results in improved correlation with the hematological outcome compared to classical *S* value dosimetry.

### Comparison of bone marrow absorbed doses

Bone marrow dosimetry estimates derived from the bone marrow models MC1, MC2, and SMIRD were compared among each other, and respective differences were further correlated with the bone lesion load, the time-integrated tumor uptake, and the time-integrated ROB retention (MATLAB Pearson’s correlation), to estimate which influencing factors of absorbed dose modeling define differences between the investigated models.

### Bone marrow dosimetry using Tc-99m-anti-granulocyte antibody scintigraphy

Pre-therapeutic Ga-68-PSMA-11 PET and Tc-99m-anti-granulocyte antibody SPECT distributions were visually compared with respect to the overlap between tumor uptake and the accumulation in the Tc-99m-anti-granulocyte antibody scintigraphy. In a second step, the Tc-99m-anti-granulocyte antibody SPECT/CT was non-rigidly co-registered to the Ga-68-PSMA-11 PET/CT data (PMOD Version 3.609) and considered during the Monte Carlo absorbed dose calculation as active bone marrow VOI (gMC3). Respective absorbed dose estimates were compared with those from model MC1 for no bone marrow displacement from the direct location of metastasis, MC2 assuming full bone marrow displacement, and SMIRD.

## Results

### Reference bone marrow dosimetry using mass-scaled organ-level *S* values

Median bone marrow absorbed dose estimates as derived via *S* values (SMIRD) were found to be 11 mGy/GBq (6–25 mGy/GBq) (Table 2). Individual bone marrow absorbed doses showed a weak positive correlation with the bone lesion load (*r* = 0.36, *p* = 0.27, *R*^2^ = 0.13). A strong positive correlation with the time-integrated ROB retention (*r* = 0.87, *p* < 0.01, *R*^2^ = 0.74) and with the time-integrated tumor uptake (*r* = 0.88, *p* < 0.01, *R*^2^ = 0.75) was found.

**Table 2:** Bone marrow absorbed dose estimates as derived either using *S* values (SMIRD), Monte Carlo simulations under the assumption of a physiological active bone marrow distribution (MC1), or Monte Carlo simulations assuming no active bone marrow at the direct location of the bone lesions (MC2)

Patient (administered activity in GBq)	SMIRD (mGy)	MC1 (mGy)	MC2 (mGy)	Bone lesion load (ml)	Time-integrated tumor uptake (GBq × s/ml)	Time-integrated ROB retention (GBq × s/ml)
P1 (3.7)	30	493	81	402	141	3
P2 (3.7)	41	109	109	33	87	4
P3 (3.7)	52	274	137	448	59	5
P4 (3.7)	63	3192	281	1123	609	4
P5 (6.0)	120	2139	515	1124	523	17
P6 (6.0)	36	22	22	50	91	6
P7 (6.0)	150	5595	635	727	1130	26
P8 (6.0)	46	1123	225	1298	159	10
P9 (6.0)	40	684	202	836	128	9
P10 (6.1)	66	782	347	1383	84	17
P11 (6.1)	36	403	206	467	64	9

Additionally, bone lesion load, time-integrated tumor uptake, and ROB retention are provided for each patient

**Fig. 2:**
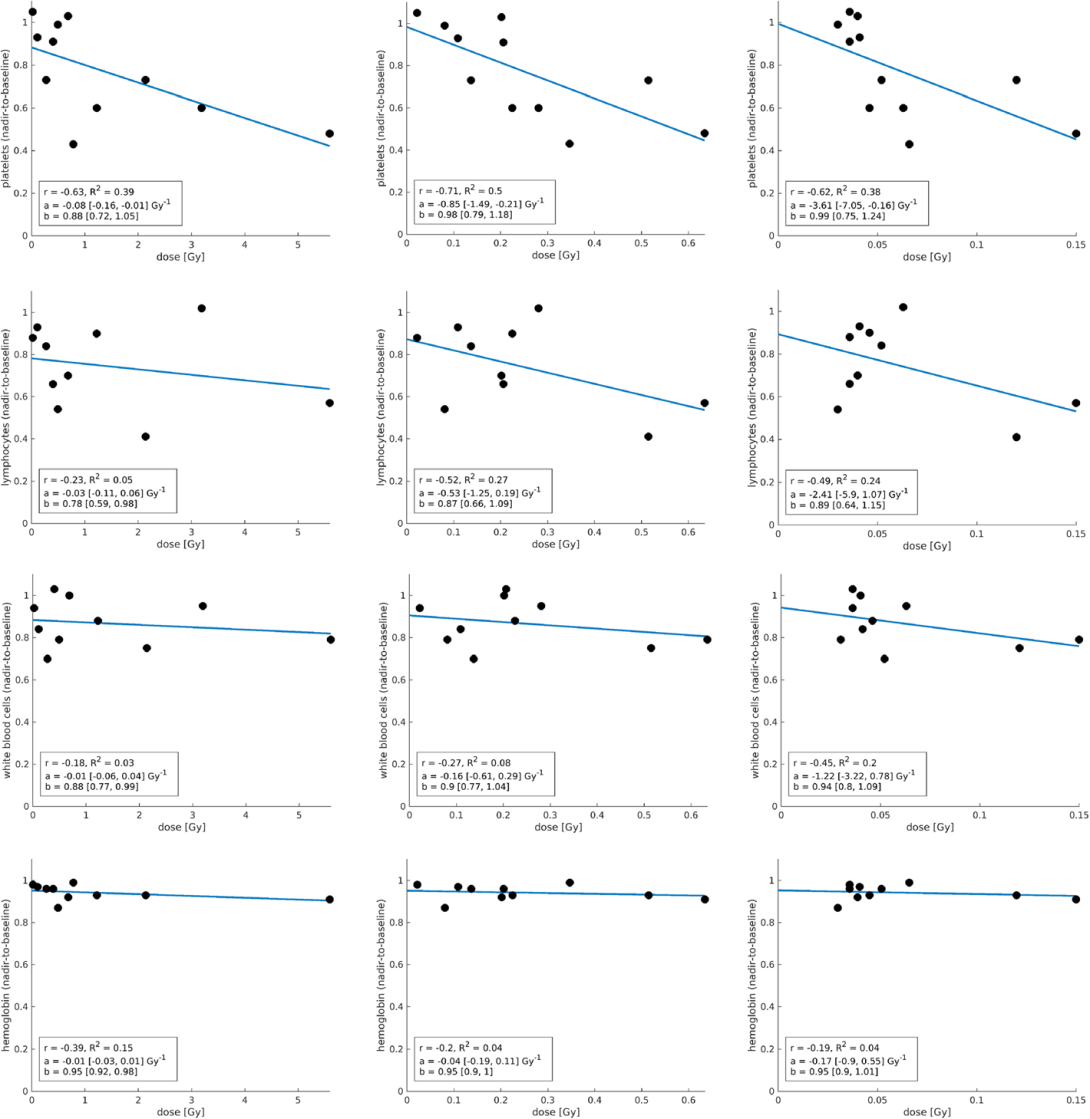
Results for the correlation analysis between the investigated models for bone marrow absorbed dose calculation and the change of blood element counts (from left to right: MC1, MC2, and SMIRD). Coefficients of the correlation model (*a* × *x* + *b*) were indicated, in combination with the 95% confidence bounds (brackets)

Analysis of blood parameters revealed a weak negative correlation of bone marrow absorbed dose estimates with the change of hemoglobin level (*r* = − 0.19, *p* = 0.60, *R*^2^ = 0.04). A moderate negative correlation was found for the change of lymphocyte counts (*r* = − 0.49, *p* = 0.15, R^2^=0.24) and total white blood cells (*r* = − 0.45, *p* = 0.20, *R*^2^=0.20), while the change of platelet counts showed a strong negative correlation (*r* = − 0.62, *p* = 0.04, *R*^2^ = 0.38) (Fig. 2).

### Monte Carlo simulation of bone marrow absorbed dose

Under the assumption of an unaltered and physiological active bone marrow distribution (MC1), median absorbed dose estimates were found to be 130 mGy/GBq (4–933 mGy/GBq) (Table 2). Further, for model MC1, a weak positive correlation with the bone lesion volume (*r* = 0.38, *p* = 0.24, *R*^2^ = 0.15), while a strong up to very strong positive correlation with the time-integrated ROB retention (*r* = 0.71, *p* = 0.01, *R*^2^ = 0.50) and time-integrated tumor uptake (*r* = 0.98, *p* < 0.01, *R*^2^ = 0.97) was observed. Analysis of blood parameters revealed a weak negative correlation with the change of lymphocyte counts (*r* = − 0.23, *p* = 0.52, *R*^2^ = 0.05), total white blood cells (*r* = − 0.18, *p* = 0.61, *R*^2^ = 0.03), and hemoglobin level (*r* = − 0.39, *p* = 0.26, *R*^2^ = 0.15). A strong negative correlation for the change of platelet counts (r = − 0.63, *p* = 0.04, *R*^2^ = 0.38) was found (Fig. 2).

For model MC2, which assumes a full displacement of active bone marrow from the direct location of each lesion, median bone marrow absorbed dose estimates were 37 mGy/GBq (4–106 mGy/GBq) (Table 2). For MC2, a moderate positive correlation with the bone lesion volume was found (*r* = 0.58, *p* = 0.06, *R*^2^ = 0.33), while a strong up to very strong positive correlation with the time-integrated tumor uptake and ROB retention was observed (*r* = 0.82, *p* < 0.01, *R*^2^ = 0.68 and *r* = 0.92, *p* < 0.01, *R*^2^ = 0.84). Concerning the blood parameters, a weak negative correlation was found for the change of hemoglobin level (*r* = − 0.20, *p* = 0.59, *R*^2^ = 0.04) and total white blood cells (*r* = − 0.27, *p* = 0.44, *R*^2^ = 0.08), while lymphocyte counts (*r* = − 0.52, *p* = 0.13, *R*^2^ = 0.27) showed a moderate negative correlation. Analysis of the change of platelet counts showed a strong negative correlation (*r* = − 0.71, *p* = 0.01, *R*^2^ = 0.50) (Fig. 2).

**Fig. 3:**
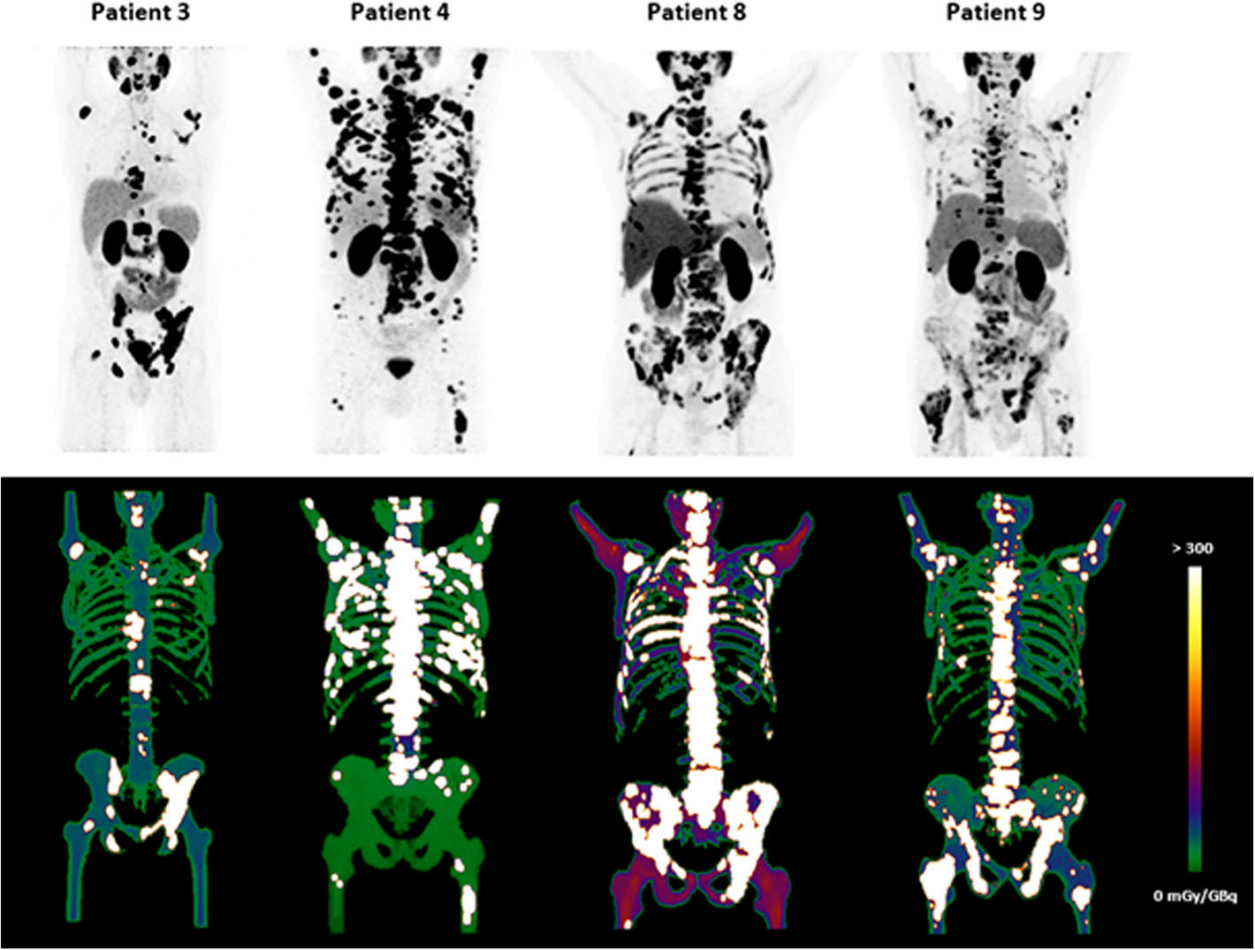
Exemplary Monte Carlo simulation results for patients 3, 4, 8, and 9. Upper row: MIP from pre-therapeutic Ga-68- PSMA-11 PET/CT; lower row: MIP for simulated absorbed dose within the bone marrow

Exemplary simulation results are provided in Fig. 3 for patients 3, 4, 8, and 9. Patients 4 and 8 present with a comparable bone lesion load; however, a clearly higher bone marrow absorbed dose was observed for patient 4 and particularly MC1, due to a fourfold higher time-integrated tumor uptake (Table 2).

### Comparison of bone marrow absorbed doses

The median ratio between models MC1 and SMIRD was a factor of 17 (1–50) (Table 3). The highest differences were observed for patients 4, 7, and 8, who showed a combination of a comparatively high bone lesion volume, a high time-integrated tumor uptake, and a high ratio of time-integrated tumor uptake to ROB retention (Table 3). The lowest differences were found for patients 2, 3, and 6. Patients 2 and 6 showed the lowest bone lesion volume (< 50 ml), while for patient 3 both a comparatively low tumor uptake and ROB retention were observed (Table 3). In general, the differences between MC1 and SMIRD were mainly driven by the time-integrated tumor uptake and the bone lesion volume, respectively (*r* = 0.77, *p* < 0.01, *R*^2^ = 0.59, and *r* = 0.60, *p* = 0.05, *R*^2^ = 0.36).

**Table 3:** Ratio of bone marrow absorbed dose estimates based on Monte Carlo simulations and *S* values

Patient	MC1/SMIRD	MC2/SMIRD	MC1/MC2
P1	17	3	6
P2	3	3	1
P3	5	3	2
P4	50	4	11
P5	18	4	4
P6	1	1	1
P7	37	4	9
P8	27	5	5
P9	17	5	3
P10	12	5	2
P11	11	6	2
Median	17	4	3

The comparison between model MC2 and SMIRD revealed a median ratio of 4 (1–6) (Table 3). The lowest difference was obtained again for patient 6, while all other patients showed a similar deviation by a factor of 3 to 6, whereupon the differences between MC2 and SMIRD mainly show a strong positive correlation with the bone lesion volume (*r* = 0.63, *p* = 0.04, *R*^2^ = 0.40).

The median ratio of MC1 to MC2 was found to be 3 (1–11), with the highest differences being found for patients 4 and 7, which both show the highest time-integrated tumor uptake (Table 3). In addition, a strong positive correlation between the ratios MC1 to MC2 with the time-integrated tumor uptake was found (*r* = 0.78, *p* < 0.01,*R*^2^ = 0.61).

### Bone marrow dosimetry using Tc-99m-anti-granulocyte antibody scintigraphy

For patients 8 and 9, bone marrow absorbed dose estimates were re-analyzed using the Tc-99m-anti-granulocyte-based active bone marrow VOI (gMC3). For patient 8, MC1 and MC2 revealed a bone marrow absorbed dose of 1223 and 225 mGy, respectively, compared to 46 mGy for SMIRD. For the Tc-99m-anti-granulocyte-based VOI, an absorbed dose of 718 mGy was found, i.e., a reduction of approximately 41% compared to MC1. The ratio of model gMC3 compared to MC2 and SMIRD was found to be 3 and 17, respectively (Table 4). For patient 9, absorbed dose estimates for MC1, MC2, and SMIRD were found to be 684, 202, and 40 mGy, respectively. Applying the Tc-99m-anti-granulocyte-based VOI yielded a bone marrow absorbed dose of 408 mGy. Thus, compared to MC1, the bone marrow absorbed dose decreased by 40%, while gMC3 produced two- and tenfold higher absorbed dose estimates compared to MC2 and SMIRD (Table 4).

**Table 4:** Comparison of bone marrow absorbed dose estimates based on Monte Carlo simulations for patients 8 and 9

Patient	MC1 (mGy)	MC2 (mGy)	gMC3 (mGy)	SMIRD (mGy)
P8	1123	225	718	46
P9	684	202	408	40

gMC3 uses the patient-specific Tc-99m-anti-granulocyte-based active bone marrow VOI. MC1 assumes a physiological active bone marrow distribution, while MC2 assumes a physiological distribution, however with displacement of active bone marrow from the direct site of metastases

**Fig. 4:**
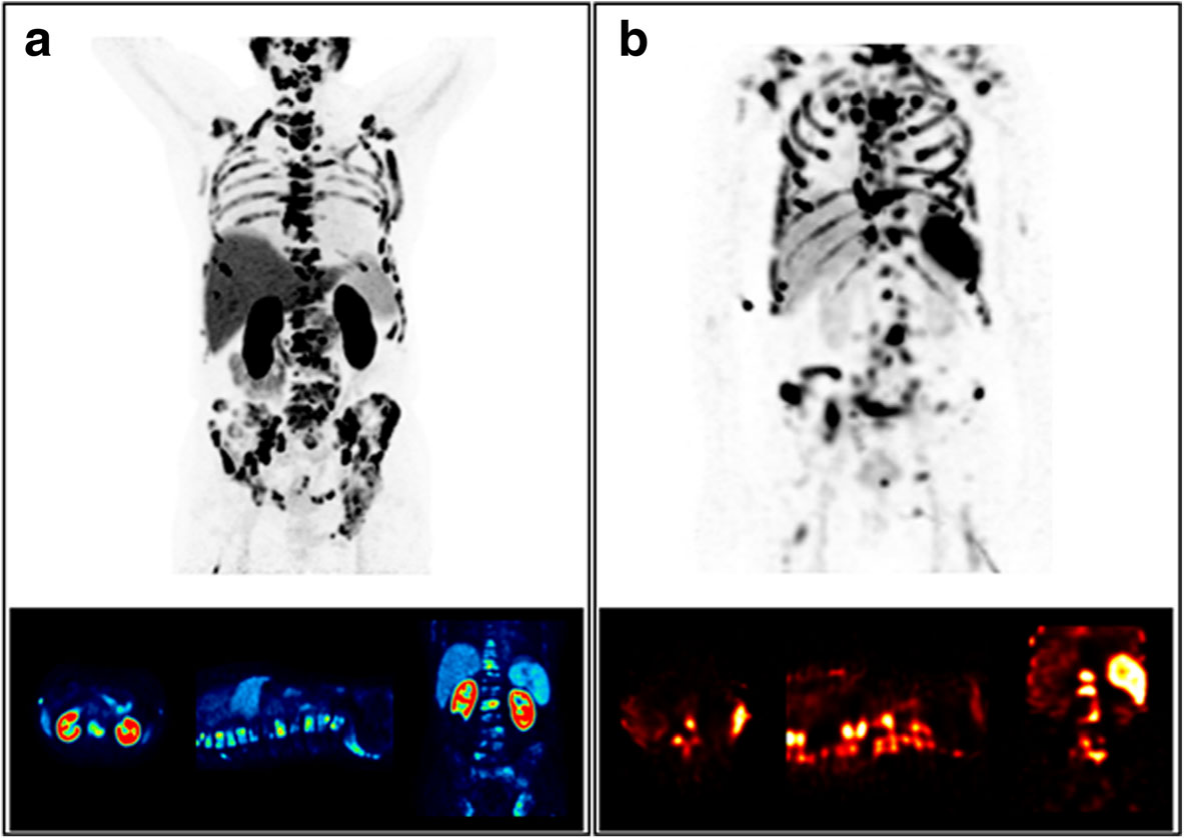
Results from Tc-99m-anti-granulocyte antibody SPECT/CT (**b**) in comparison to the Ga-68-PSMA-11 PET/CT (**a**) for patient 8

**Fig. 5:**
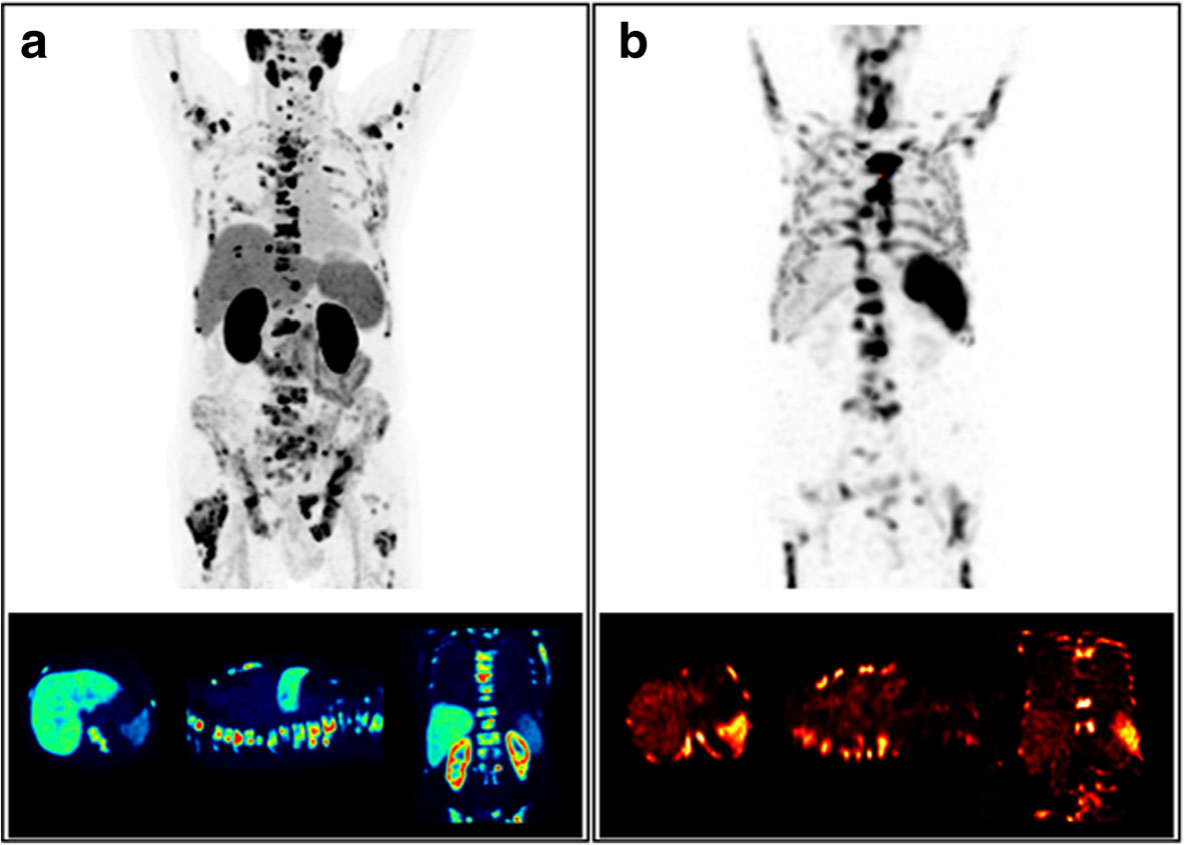
Results from Tc-99m-anti-granulocyte antibody SPECT/CT (**b**) in comparison to the Ga-68- PSMA-11 PET/CT (**a**) for patient 9

Furthermore, visual interpretation of Ga-68-PSMA-11 PET and Tc-99m-anti-granulocyte antibody scintigraphy indicates a low overlap between accumulation patterns and a displacement of active bone marrow from metastatic lesions for both investigated patients (Figs. 4 and 5).

## Discussion

The bone marrow is potentially the most critical organ and most limiting factor of the therapeutic window during Lu-177-PSMA therapy of mCRPC patients, as those patients are usually heavily pre-treated and often present with a considerable bone lesion load [14]. In a study of Rahbar et al. with 145 patients and an average administered activity of 5.9 GBq per cycle, hematoxicity showed the highest incidence for all grades as well as for grade 3–4 events [36]. Thus, bone marrow dosimetry is highly recommended in those patients. However, for bone marrow dosimetry to become predictive for hematoxicity, all relevant patient-specific parameters must be considered. These include the patient-specific activity accumulation over time, the anatomical characteristics, an appropriate localization of the bone marrow target region, and, not least, pre-therapies and the patient-specific disease and hematological baseline status.

This study focuses on three relevant issues: First, we developed an approach for Monte-Carlo-based absorbed dose calculations, which can fully consider the patient-specific 3D activity and anatomical characteristics in contrast to the classical *S* value dosimetry. Second, we compared the effect of different models for active bone marrow localization during Monte-Carlo-based bone marrow dosimetry. The latter aspect is especially linked to the question whether patient-specific active bone marrow localization, e.g., via Tc-99m-anti-granulocyte scintigraphy, might be beneficial to avoid bone marrow toxicity. Third, we investigated whether for the patient cohort under study, Monte-Carlo-based absorbed dose calculation shows an improved correlation with the change of hematological parameters, and whether fully patient-specific bone marrow dosimetry can potentially provide an improved prediction for hematoxicities.

Although the number of patients investigated in this study is low, our preliminary results already indicate a large range between bone marrow absorbed dose estimates for Monte Carlo and *S* value calculations. The model-specific correlation of bone marrow absorbed dose estimates with segmented bone lesion volume, time-integrated tumor uptake, and ROB retention, as performed during this study, supports that multiple patient-specific factors should be taken into consideration to reduce the uncertainty of bone marrow dosimetry. SMIRD-based bone marrow dosimetry mainly accounts for the time-integrated ROB and lesion uptake characteristics; however, respective absorbed doses revealed only a weak correlation with the patient-specific and highly heterogeneous 3D bone lesion distribution. For MC2-based absorbed dose estimates, a strong and significant correlation with tumor uptake, ROB retention, and bone lesion volume was found, while bone marrow absorbed dose calculations during model MC1 are clearly dominated by the time-integrated tumor uptake. Results from correlation analysis support that also for 3D-based absorbed dose calculation, the total bone lesion volume is not the only influencing factor of the bone marrow absorbed dose, and risk patients should be stratified according to multiple parameters.

With respect to the absolute values, patient-specific Monte-Carlo-based calculations resulted in higher bone marrow absorbed doses than the classical *S* value approach. For both models, MC2 and SMIRD, bone marrow absorbed dose estimates were well below the typically applied threshold of 2 Gy [19]. Although for MC2 the absorbed dose estimates were on median fourfold higher than those achieved with the reference model SMIRD, the deduction that multiple therapy cycles are applicable for these patients without the risk of severe marrow toxicities seems reasonable, at least with regard to the current dose limit. However, the assumption of full displacement of active bone marrow, as included in model MC2, might not be a priori justified in each patient and might lead to an underestimation of the bone marrow absorbed dose. For model MC1, assuming a physiological and unaltered active bone marrow distribution, median absorbed doses of 130 mGy/GBq were found with on median 17-fold and 3-fold higher absorbed dose estimates compared to SMIRD and MC2, respectively. According to dosimetry using model MC1, patients 4, 5, 7, and 8 would have received a bone marrow absorbed dose close to or in some cases even strongly exceeding the 2 Gy limit, with the consequent risk for severe marrow toxicities in these patients. However, none of the patients presented severe marrow damage, even after multiple therapy cycles. This observation questions the general applicability and significance of models like MC1, which do not account for bone marrow displacement by tumor lesions, especially in the presence of a heavy skeletal tumor burden.

Visual analysis of Tc-99m-anti-granulocyte antibody SPECT scans supports this thesis, as it indicates a displacement of active bone marrow from the direct location of bone lesions for both exemplarily investigated patients. In these patients, the re-analysis of bone marrow absorbed dose estimates using the individual Tc-99m-anti-granulocyte-based active bone marrow VOIs resulted in a clear absorbed dose reduction of approximately 40% compared to MC1, although the absorbed dose values were still higher than those from MC2. However, both the Ga-68-PSMA-11 PET and the Tc-99m-anti-granulocyte antibody SPECT have a finite resolution, which intrinsically results in a certain artificial overlap of both activity distributions and thus in an increased absorbed dose estimate compared to MC2. Furthermore, co-registration between both modalities is in general not perfect, particularly for challenging regions such as the ribs or the sternum, which might additionally cause a local overlap between tumor and bone marrow accumulation. Both finite spatial resolution and imperfect co-registration interfere with a potential incomplete active bone marrow displacement. Despite the additional complexity introduced by the finite resolution of the involved imaging systems and by the imperfect co-registration, the utilization of the additional image data from the Tc-99m-anti-granulocyte antibody scintigraphy may improve individualized bone marrow dosimetry by providing a realistic upper limit for the bone marrow absorbed dose. For patient 8, the usage of a Tc-99m-anti-granulocyte-based VOI resulted in a reduction of a total bone marrow absorbed dose from 1.1 Gy (MC1) to an expected maximum dose of 0.7 Gy. It may be hypothesized that the typically applied upper limit for the bone marrow absorbed dose of 2 Gy is too high for patients with advanced cancer disease, extensive skeletal tumor burden, and potentially decreased hematological function due to various pre-therapies. Despite the relatively small additional bone marrow absorbed dose (approx. 10 mSv for 400 MBq), Tc-99m-anti-granulocyte antibody scintigraphy might be justified in such patients. Studies based on a larger patient cohort are needed to identify, whether image-based active bone marrow localization in combination with Monte-Carlo-based absorbed dose calculation really improves the correlation between bone marrow dosimetry and hematoxicities, and may therefore be suitable to guide therapy planning in future workflows. Still, the comparison between models MC1 and MC2 demonstrates that the a priori unknown patient-specific active bone marrow distribution results in a large uncertainty of the bone marrow absorbed doses, even if Monte Carlo techniques are applied for absorbed dose modeling. Further, future studies should be performed to find an appropriate threshold for the bone marrow absorbed dose for Lu-177-PSMA therapy [14].

The resolution of non-invasive active bone marrow localization could be further improved by MRI bone marrow localization, which would also be beneficial if even small additional contributions to the bone marrow absorbed dose must be avoided [24, 25]. However, in clinical daily routine, the localization of active bone marrow in a large part of the patient body should be feasible with acceptable measurement and processing time, and it should be available for several patients per week. Further, a reduction of processing complexity and effort accompanied by a potential enhancement of the accuracy of the derived information is desirable. This could be achieved, for instance, by employing a standardized method for patient positioning over multiple scans at the same or at different imaging modalities via patient-adaptable storage matrasses, which facilitates image co-registration [37].

To derive a patient-specific activity template from the Ga-68-PSMA-11 PET instead from the Lu-177-SPECT reduces issues of spatial resolution during absorbed dose calculation. Further, for the patients considered in this study, only SPECT acquisitions of the abdomen were available, while for bone marrow dosimetry, the lesion distribution in the overall body is important. Using the Ga-68-PSMA-11 PET to model the 3D activity accumulation during therapy is an approximation and only applicable if the delay between PET acquisition and therapy is small (on average 2.5 weeks in this study) and if there is no change in the overall lesion load. To switch to a fully Lu-177-based activity template, further investigations are desired to improve the spatial resolution of the Lu-177 imaging and to enable a fast whole-body Lu-177-SPECT acquisition. Filling a fixed patient-specific activity VOI template with the respective segmented VOI activities from sequential Lu-177 imaging reduces co-registration errors, which could otherwise lead to an artificially increased bone marrow absorbed dose.

The analysis of blood parameters revealed a significant (*p* < 0.05) and strong negative correlation only for the change of platelet counts, irrespective of the exact bone marrow model used. The highest correlation was obtained with model MC2, which includes the assumption of full displacement of active bone marrow from the direct site of the bone lesions. To exploit the potential and impact of bone marrow dosimetry for therapy planning, a more comprehensive investigation of the correlation of the change of blood element counts with bone marrow absorbed dose estimates is desired. For this purpose, blood analysis should consider a higher number of patients and a prolonged time period. Both the baseline hematological status and its course after therapy are known to be affected by various parameters, such as pre-therapies or total lesion volume [17, 38–40]. Thus, a patient stratification as for example proposed by Walrand et al. is mandatory, if the correlation between bone marrow absorbed doses and hematological response to therapy shall be analyzed [40]. So far, the correlation of bone marrow absorbed doses and blood parameters for different Lu-177-based radioligand therapies was assessed using *S* value-based methods. Svensson et al. observed moderate and significant correlations for the decrease of hemoglobin level, total white blood cells, and platelet counts for Lu-177 PRRT and for 46 investigated patients [17]. By contrast, Forrer et al. found no correlation between the decrease of platelet counts and bone marrow absorbed dose estimates for Lu-177 PRRT based on 15 patients and monitoring of hematological function until 6 weeks after treatment [18].

In clinical routine, the application of Monte Carlo simulations for dose calculations may be too time-consuming, especially if the simulation has to consider a large part of the patient body and small voxels (e.g., 0.05–0.001 ccm). However, Monte Carlo simulations can be made feasible with computing clusters. In this way, results with a high statistical validity could be obtained in 1 day, which is acceptable with respect to the time gap between successive cycles of radioligand therapy. An intermediate method for fast 3D dosimetry within minutes, which compromises the consideration of Monte Carlo techniques and computational effectiveness, is the application of Monte-Carlo-based dose kernels [41, 42]. Further investigations of this approach may be advisable to prospectively facilitate improved clinical dosimetry for monitoring and planning of radioligand therapies.

In this work, we introduced a weighting-based model to represent the different compartments of active and inactive bone marrow and hard bone. This model represents a reasonable simplification for the application to clinical routine imaging data, which have a spatial resolution above the characteristic size of the bone marrow microstructure. Hybrid Monte-Carlo-based models that alternate between macroscopic models of the overall patient anatomy and detailed microscopic models of the skeletal system are time-consuming but might further improve bone marrow absorbed dose estimates, at least for the understanding of important mechanisms to assess risk factors for marrow toxicities [34, 43].

## Conclusion

Monte-Carlo-based bone marrow absorbed doses were found to be significantly increased compared to those derived from classical *S* value dosimetry. Particularly, a large spread between Monte-Carlo-based and *S* value bone marrow absorbed doses was observed, which implies a large uncertainty, especially for *S* value dosimetry due to the lack of an appropriate consideration of the patient-specific highly heterogeneous 3D lesion distribution. However, even for Monte-Carlo-based bone marrow dosimetry, the a priori unknown patient-specific active bone marrow distribution produces a large uncertainty of bone marrow absorbed doses. Assuming a co-localization between active bone marrow and all lesions (MC1) is hypothesized to lead to too exaggerated absorbed dose values (> 2 Gy per cycle for 27% of investigated cycles), as these values were not in concordance with the observation of severe hematological toxicities. Simultaneously, the a priori assumption of a full displacement of active bone marrow for each patient (MC2) might underestimate the patient-specific absorbed dose. Patient-specific image-based active marrow localization, as performed for a small subgroup of patients, yielded to intermediate bone marrow absorbed doses compared to MC1 and MC2, although issues of co-registration and finite image resolution might interfere with incomplete active bone marrow displacement. Future studies based on a larger patient cohort are recommended, to particularly determine whether patient-specific active bone marrow localization in combination with Monte-Carlo-based absorbed dose modeling can improve the prediction of hematoxicities and thus enables to exploit the full therapeutic window of Lu-177-PSMA therapy. Preliminary results showed a significant and strong correlation between platelet decrease and bone marrow absorbed doses, irrespective of the exact dosimetry model; however, highest correlation was observed for MC2.

## Data Availability

Please contact the corresponding author for data request.

## Abbreviations

AC-CT: Low dose attenuation correction CT
BM: Bone marrow
CT: Computed tomography
ICRP: Internal Commission on Radiological Protection
MC: Monte Carlo
mCRPC: Metastasized castration-resistant prostate cancer
MIRD: Medical Internal Radiation Dose (Committee)
PET: Positron emission tomography
PRRT: Peptide receptor radionuclide therapy
PSMA: Prostate-specific membrane antigen
RADAR: Radiation Dose Assessment Resource
ROB: Remainder of the body
SPECT: Single-photon emission computed tomography
VOI: Volume of interest
